# Selective Upregulation of Transcripts for Six Molecules Related to T Cell Costimulation and Phagocyte Recruitment and Activation among 734 Immunity-Related Genes in the Brain during Perforin-Dependent, CD8^+^ T Cell-Mediated Elimination of Toxoplasma gondii Cysts

**DOI:** 10.1128/mSystems.00189-20

**Published:** 2020-04-14

**Authors:** Jenny Lutshumba, Eri Ochiai, Qila Sa, Namrata Anand, Yasuhiro Suzuki

**Affiliations:** a Department of Microbiology, Immunology and Molecular Genetics, University of Kentucky College of Medicine, Lexington, Kentucky, USA; University of California, San Francisco

**Keywords:** CD8^+^ cytotoxic T cells, perforin, T cell invasion, coactivation molecules, phagocytes, chemokine receptor, costimulatory molecules, large-scale gene expression profile

## Abstract

We recently found that an invasion of CD8^+^ cytotoxic T cells into tissue cysts of Toxoplasma gondii initiates an elimination of the cysts in association with an accumulation of microglia and macrophages. In the present study, we compared mRNA levels for 734 immune-related genes in the brains of infected SCID mice that received perforin-sufficient or -deficient CD8^+^ immune T cells at 3 weeks after infection. At 7 days after the T cell transfer, mRNA levels for only six genes were identified to be greater in the recipients of the perforin-sufficient T cells than in the recipients of the perforin-deficient T cells. These six molecules included two T cell costimulatory molecules, inducible T cell costimulator receptor (ICOS) and its ligand (ICOSL); two chemokine receptors, C-X-C motif chemokine receptor 3 (CXCR3) and CXCR6; and two molecules related to an activation of microglia and macrophages, interleukin 18 receptor 1 (IL-18R1) and chitinase-like 3 (Chil3). Consistently, a marked reduction of cyst numbers and upregulation of ICOS, CXCR3, CXCR6, IL-18R1, and Chil3 mRNA levels were also detected when the perforin-sufficient CD8^+^ immune T cells were transferred to infected SCID mice at 6 weeks after infection, indicating that the CD8^+^ T cell-mediated protective immunity is capable of eliminating mature T. gondii cysts. These results together suggest that ICOS-ICOSL interactions are crucial for activating CD8^+^ cytotoxic immune T cells to initiate the destruction of T. gondii cysts and that CXCR3, CXCR6, and IL-18R are involved in recruitment and activation of microglia and macrophages to the T cell-attacked cysts for their elimination.

**IMPORTANCE** T. gondii establishes a chronic infection by forming tissue cysts, which can grow into sizes greater than 50 μm in diameter as a consequence of containing hundreds to thousands of organisms surrounded by the cyst wall within infected cells. Our recent studies using murine models uncovered that CD8^+^ cytotoxic T cells penetrate into the cysts in a perforin-dependent manner and induce their elimination, which is accompanied with an accumulation of phagocytic cells to the T cell-attacked target. This is the first evidence of the ability of the T cells to invade into a large target for its elimination. However, the mechanisms involved in anticyst immunity remain unclear. Immune profiling analyses of 734 immune-related genes in the present study provided a valuable foundation to initiate elucidating detailed molecular mechanisms of the novel effector function of the immune system operated by perforin-mediated invasion of CD8^+^ T cells into large targets for their elimination.

## Abstract (precis)

T. gondii establishes a chronic infection by forming tissue cysts, which can grow into sizes greater than 50 μm in diameter as a consequence of containing hundreds to thousands of organisms surrounded by the cyst wall within infected cells. Our recent studies using murine models uncovered that CD8^+^ cytotoxic T cells penetrate into the cysts in a perforin-dependent manner and induce their elimination, which is accompanied with an accumulation of phagocytic cells to the T cell-attacked target. This is the first evidence of the ability of the T cells to invade into a large target for its elimination. However, the mechanisms involved in anticyst immunity remain unclear. Immune profiling analyses of 734 immune-related genes in the present study provided a valuable foundation to initiate elucidating detailed molecular mechanisms of the novel effector function of the immune system operated by perforin-mediated invasion of CD8^+^ T cells into large targets for their elimination.

## INTRODUCTION

Toxoplasma gondii is an obligate intracellular protozoan parasite capable of infecting warm-blooded animals, including mammals and birds. During the acute stage of infection, tachyzoites, the acute-stage form, invade host cells in various tissues and proliferate within the host cells (1–3). The presence of tachyzoites leads to a surge in gamma interferon (IFN-γ)-mediated protective immune responses to control their proliferation (4). IFN-γ activates various types of cells, both phagocytic and nonphagocytic, to inhibit intracellular tachyzoite growth (5, 6). However, tachyzoites convert into bradyzoites and form tissue cysts to establish a chronic infection (2, 7, 8). T. gondii cysts can grow to more than 50 μm in diameter by folding hundreds to thousands of bradyzoites surrounded by the cyst wall within infected cells (2, 7, 8). Approximately 30% of the world’s human population is estimated to be chronically infected with T. gondii (3). Immunocompetent individuals infected with T. gondii are usually asymptomatic, but it has recently been shown that this chronic infection is associated with increased incidence of brain cancers (9, 10) and with higher mortality in these cancer patients (11). In immunocompromised individuals such as those with HIV infection or transplanted organs, reactivation of the chronic T. gondii infection can occur and lead to serious toxoplasmic encephalitis (3).

Although there are treatments to alleviate the acute phase of T. gondii infection by inhibiting tachyzoite proliferation, there are no drugs available to eradicate tissue cysts of the parasite. It was generally considered that this chronic phase is persistent due to lack of a capability in the immune system to recognize or eliminate T. gondii cysts located within infected cells. However, we recently discovered that an adoptive transfer of CD8^+^ immune T cells isolated from infected wild-type (WT) mice, which are genetically resistant to the infection, was able to markedly reduce numbers of cysts in the brains of infected, immunodeficient mice, such as SCID and nude, when the recipient animals received the T cells after having developed large numbers of cysts in their brains (12, 13). The removal of cysts by CD8^+^ immune T cells was identified to be perforin dependent (12, 13). Perforin plays an important role in the cytotoxic activity of CD8^+^ T cells. Perforin secreted from the cytotoxic T cells binds the surface of the cell membrane of the target cells and forms pores in the cell membrane, which is required for their cytotoxic activity. Notably, our studies revealed that the cytotoxic T cells penetrate into T. gondii cysts in a perforin-dependent manner to induce morphological deterioration and destruction of the cysts (13).

During the perforin-dependent, CD8^+^ T cell-mediated removal of T. gondii cysts, microglia and inflammatory macrophages accumulated around and within the morphologically deteriorated cysts and destroyed the cysts (12, 13). Since many bradyzoites present within the destroyed cysts were found to be located within these accumulated phagocytes (13), these cells most likely represent scavenger cells that eliminate the parasite when the CD8^+^ T cells attack the cysts and initiate the anticyst immune process. However, the molecular mechanisms by which CD8^+^ cytotoxic T cells and the phagocytes collaborate to eliminate the cysts remain to be determined.

In the present study, to obtain an unbiased overview of the immune responses involved in the collaboration of CD8^+^ cytotoxic T cells and phagocytes to eliminate T. gondii cysts, we performed an analysis using a NanoString panel to measure mRNA levels of 734 genes involved in the immune responses in the brains of immunodeficient SCID mice following an adoptive transfer of perforin-sufficient and perforin-deficient CD8^+^ immune T cells obtained from infected WT and perforin knockout (Prf1^−/−^) mice. This immune profiling analysis revealed that the mRNA levels for only six genes, inducible T cell costimulator receptor (ICOS), ICOS ligand (ICOSL), C-X-C motif chemokine receptor 3 (CXCR3), CXCR6, interleukin 18 receptor 1 (IL-18-R1), and chitinase-like 3 (Chil3), among the 734 immunity-related genes tested were upregulated only when the CD8^+^ T cells expressed perforin. Consistently, further analyses using real-time reverse transcriptase PCR (RT-PCR) on the brains of SCID mice that had received CD8^+^ T cells from uninfected or infected WT mice revealed that four genes, ICOS, CXCR3, CXCR6, and IL-18R1, among the 6 genes were significantly upregulated in the brain of the immune T cell recipients compared to normal CD8^+^ T cell recipients in which the cyst elimination did not occur. ICOS is one of T cell costimulation molecules (14, 15). CXCR3, CXCR6 (16–19), and IL-18R1 are the molecules involved in recruitment and/or activation of microglia and macrophages (20, 21). Therefore, the results of the present study suggest that ICOS plays an important role in activating CD8^+^ cytotoxic T cells to initiate attacking T. gondii cyst-holding cells and that CXCR3, CXCR6, and IL-18R1 are involved in recruiting and activating the phagocytes corresponding to the T cell-attacked cysts for eradicating bradyzoites located within the cysts for their elimination.

## RESULTS

### Expression levels of mRNA determined for six molecules related to T cell stimulation and recruitment and activation of microglia and macrophages upregulated during the perforin-dependent elimination of T. gondii cysts by CD8^+^ T cells.

**FIG 1:**
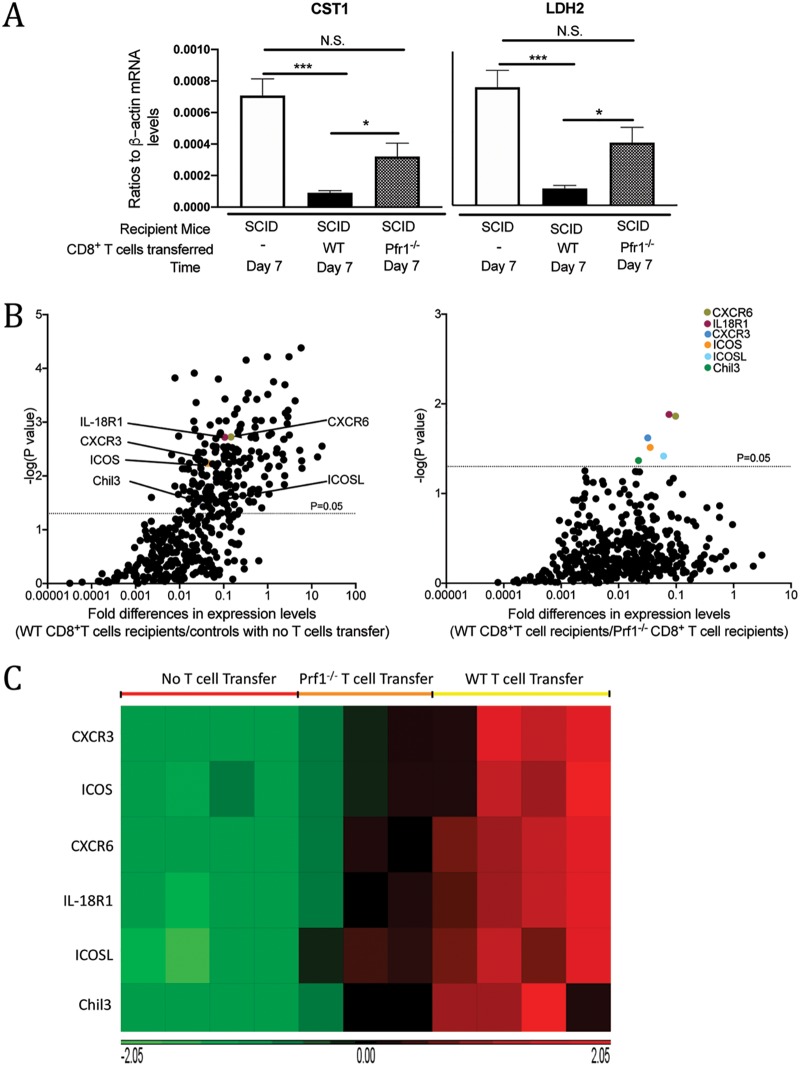
Differences in mRNA expression levels for 734 genes related to the immune responses in the brains of T. gondii-infected SCID mice that had received WT or Prf1^−/−^ CD8^+^ immune T cells. SCID mice were infected with 20 cysts of the ME49 strain of T. gondii and treated with sulfadiazine beginning at 9 days after the infection to induce formation of tissue cysts of the parasite in their brains. At 3 weeks after infection, animals were injected intravenously from a tail vein with CD8^+^ immune T cells (2.1 × 10^6^ cells) from infected WT or Pfr1^−/−^ mice. As a control, one group of infected SCID mice did not receive any T cells. Seven days later, their brains were harvested for extracting RNA. (A) Significantly smaller amounts of mRNA for bradyzoite (cyst)-specific CST1 and LDH2 were detected in the brains of infected SCID mice that had received WT CD8^+^ immune T cells than those that had received Prf1^−/−^ CD8^+^ immune T cells and the control animals that had received no T cells. *, *Pc *< 0.05; ***, *Pc *< 0.001. N.S., not significant. (B) Volcano plot indicating that the mRNA levels for only 6 genes, *Icos*, *IcosL*, *Cxcr3*, *Cxcr6*, *Il18r1*, and *Chil3*, were significantly higher in the recipients of WT CD8^+^ immune T cells than in the recipients of Prf1^−/−^ CD8^+^ T cells (right panel). Horizontal dotted lines in the volcano plots represent the threshold line for *P = *0.05. (C) Heat map visualizing higher expression of the six genes in the WT CD8^+^ T cell recipients than the Prf1^−/−^ CD8^+^ T cell recipients and no-T cell transfer controls. There were 3 to 5 mice in each experimental group.

We recently identified that perforin-dependent activity of CD8^+^ T cells is capable of removing preexisting tissue cysts of T. gondii in collaboration with microglia and macrophages (12, 13). To begin analyzing the mechanisms of the perforin-dependent anticyst immune process, we first performed a screening assay using a NanoString panel, which covers 734 molecules related to innate and T cell immunity, to identify the genes whose expression levels are upregulated during the cytotoxic T cell-dependent cyst elimination process. SCID mice were infected and treated with sulfadiazine to control tachyzoite proliferation and establish chronic infection in their brains, and 3 weeks later, the animals received CD8^+^ immune T cells from chronically infected WT or Prf1^−/−^ mice. As an additional control, a group of infected SCID mice did not receive any T cells. We first confirmed an occurrence of perforin-dependent cyst removal by measuring expression levels of mRNA for bradyzoite (cyst)-specific molecules CST1 and LDH2 in the brains of the recipient animals by real-time RT-PCR. At 7 days after the T cell transfer, the amounts of mRNA for CST1 and LDH2 were markedly lower in the brains of SCID mice that had received WT CD8^+^ immune T cells than in those of the mice that had received Prf1^−/−^ T cells and of the control animals that had received no T cells (corrected *P* [*Pc*] < 0.05 and *Pc *< 0.001, respectively, Fig. 1A). In contrast, the mRNA levels for each of these bradyzoite-specific molecules in the brains of the recipients of Prf1^−/−^ CD8^+^ T cells did not significantly differ from those in the brains of the controls without the T cell transfer (Fig. 1A).

Next, mRNA levels for 734 genes related to innate and T cell immune responses were compared between the recipients of WT CD8^+^ immune T cells and the recipients of Prf1^−/−^ CD8^+^ T cells as well as the control mice that received no T cells. The volcano plot showing results of comparisons between the WT CD8^+^ T cell recipients and the no-T cell transfer control group identified 297 genes whose mRNA levels were greater in the former than the latter (Fig. 1B, left panel). In contrast, in comparisons between the WT CD8^+^ T cell recipients and Prf1^−/−^ CD8^+^ T cell recipients, mRNA levels for only 6 molecules, ICOS, ICOSL, CXCR3, CXCR6, IL-18R1, and Chil3, were significantly greater in the brains of the former than in those of the latter (Fig. 1B, right panel). The mRNA levels for these 6 molecules were also greater in the WT CD8^+^ T cell recipients than in the no-T cell transfer controls as well (Fig. 1B, left panel). The differences in relative expression levels of mRNA for these 6 genes between the three experimental groups are visualized in a heat map (Fig. 1C). These results suggest that these six molecules play important roles in the perforin-dependent, CD8^+^ T cell-mediated immune process to eliminate T. gondii cysts from the brain.

### Costimulatory molecules were upregulated in their mRNA levels in association with the CD8^+^ T cell-mediated removal of T. gondii cysts.

**FIG 2:**
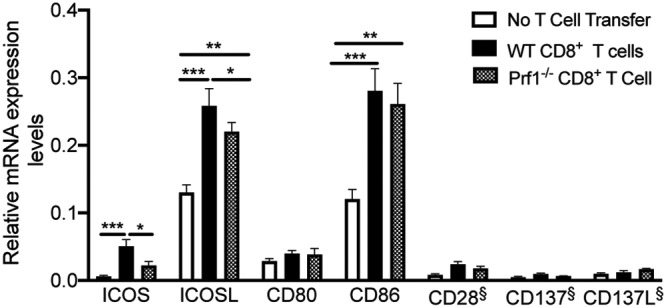
Differences in amounts of mRNA for T cell costimulatory molecules ICOS, ICOSL, CD137, CD137L, CD28, CD80, and CD86 in T. gondii-infected SCID mice that had received WT or Prf1^−/−^ CD8^+^ immune T cells and in control animals that had received no T cells. SCID mice were infected with 20 cysts of the ME49 strain of T. gondii and treated with sulfadiazine beginning at 9 days after the infection to develop tissue cysts of the parasite in their brains. Animals were injected intravenously from a tail vein with CD8^+^ immune T cells (2.1 × 10^6^ cells) from infected WT or Pfr1^−/−^ mice at 3 weeks after infection. As a control, one group of infected SCID mice did not receive any T cells. The data represent results determined 7 days after the T cell transfer. There were 3 to 5 mice in each experimental group. Data shown are means ± standard errors of the means (SEM). *, *P < *0.05; **, *P < *0.01; ***, *P < *0.001. §, below the background value of the assay, which is the average of data from the negative-control transcripts.

Among the 6 molecules whose expression levels were selectively upregulated during the perforin-dependent anticyst immune process, ICOS and ICOSL are those involved in T cell costimulation. Therefore, we compared the mRNA levels for these two molecules with those for the other T cell costimulatory molecules, CD137 (4-1BB, Tnfsf9), CD137 ligand (CD137L [4-1BBL, Tnfrsf9]), CD28, CD80, and CD86 (Fig. 2). Consistent with the results from the volcano plot analysis shown in Fig. 1B, the amounts of mRNA for both ICOS and ICOSL in the brains of the infected SCID mice that had received WT CD8^+^ immune T cells were significantly greater than in those of the animals that had received Prf1^−/−^ CD8^+^ T cells as well as in those of the control animals that had received no T cells (*P < *0.05 and *P < *0.001, respectively, Fig. 2). In contrast, these differences between groups were not detected on the mRNA level for CD80 and CD86, although the mRNA levels for these molecules were detectable in these animals (Fig. 2). The expression levels of CD86 were upregulated in the recipients of CD8^+^ T cells regardless of the presence or absence of perforin in the T cells (Fig. 2). The mRNA levels for CD137, CD137L, and CD28 were lower, and their mRNA levels were below the background value of the assay, which represents the average of data from the negative-control transcripts (Fig. 2). These results indicate that ICOS and ICOSL were unique among the seven T cell-costimulatory molecules and that their mRNA levels increased only when the perforin-dependent effector function of CD8^+^ T cells operated against T. gondii cysts. Therefore, the ICOS-ICOSL interactions appear to play an important role in activating anticyst activities of CD8^+^ cytotoxic T cells to initiate an elimination of T. gondii cysts.

The interactions between CD137 and CD137L are known to be important for CD8^+^ T cell activation in general. The finding of low mRNA levels for CD137 does not exclude the possibility that the CD137-CD137L interactions are also involved in the activation of anticyst activity of CD8^+^ cytotoxic T cells in the recipients of WT immune T cells. Since the CD8^+^ T cells transferred into infected SCID mice in the present study were those that had already been primed with T. gondii antigens in the infected donors, these primed CD8^+^ T cells were most likely already expressing sufficient levels of CD137 on their surface such as are required for their secondary responses when transferred into the recipients, and this could be a reason why mRNA levels for CD137 remained low in the recipients of the WT CD8^+^ immune T cells.

### Chemokine receptors were upregulated in their mRNA levels in association with the CD8^+^ T cell-mediated removal of T. gondii cysts.

**FIG 3:**
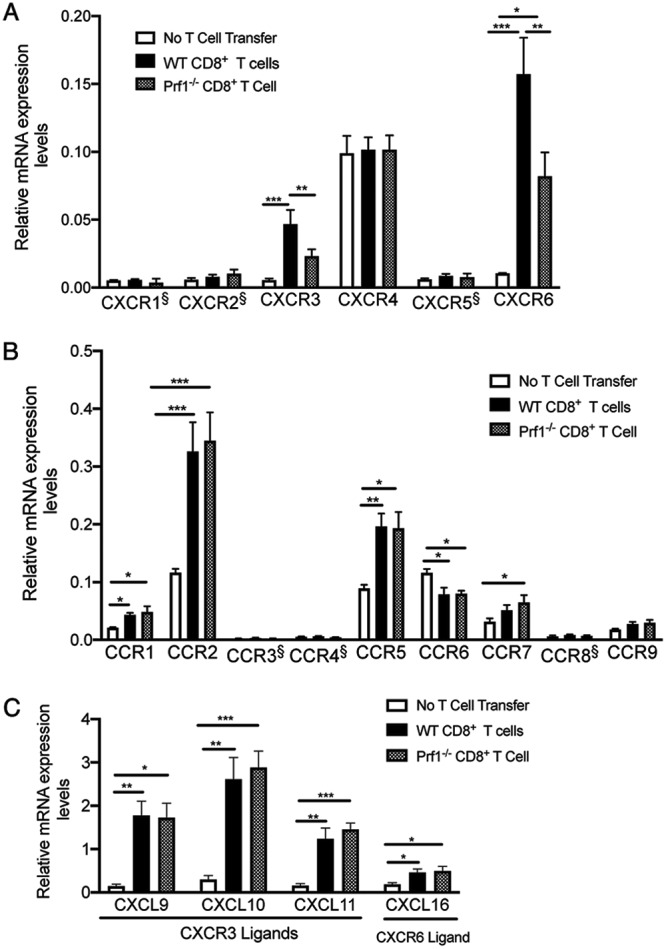
Differences in amounts of mRNA for 15 chemokine receptors, CXCR1 to CXCR6 (A) and CCR1 to CCR9 (B), and ligands for CXCR3 and CXCR6 (C) in T. gondii-infected SCID mice that had received WT or Prf1^−/−^ CD8^+^ immune T cells and in control animals that had received no T cells. SCID mice were infected with 20 cysts of the ME49 strain of T. gondii and treated with sulfadiazine beginning at 9 days after the infection to develop tissue cysts of the parasite in their brains. Animals were injected intravenously from a tail vein with CD8^+^ immune T cells (2.1 × 10^6^ cells) from infected WT or Pfr1^−/−^ mice at 3 weeks after infection. As a control, one group of infected SCID mice did not receive any T cells. The data represent results determined 7 days after the T cell transfer. There were 3 to 5 mice in each experimental group. Data shown are means ± SEM. *, *P < *0.05; **, *P < *0.01; ***, *P < *0.001. §, below the background value of the assay, which is the average of data from the negative-control transcripts.

Interactions between secreted chemokines and chemokine receptors expressed on the surface of immune cells play critical roles in mediating migration and recruitment of the immune cells into organs and to specific locations within the tissues. Those six molecules, whose expression levels were selectively upregulated during the perforin-dependent anticyst immune process, include two chemokine receptors, CXCR3 and CXCR6. Their mRNA levels along with the other 13 chemokine receptors, including 4 additional CXCR and 9 C-C motif chemokine receptors (CCR), covered by the NanoString assay are shown in Fig. 3A.

Among the four CXCR chemokine receptors other than CXCR3 and CXCR6, high levels of mRNA for CXCR4 were detected in all three groups of infected SCID mice with and without T cell transfer, and there were no differences in its expression levels among the groups (Fig. 3A). Expression levels for CXCR1, CXCR2, and CXCR5 were low and below the background value of the assay in all three groups (Fig. 3A). Among the nine CCR chemokine receptors tested, increased levels of mRNA for CCR1, CCR2, CCR5, and CCR7 were detected in the recipients of either WT or Prf1^−/−^ CD8^+^ immune T cells compared to the control animals without the T cell transfer (Fig. 3B). However, their expression levels did not differ between the recipients of the WT T cells and those receiving the Prf1^−/−^ T cells (Fig. 3B). Expression levels of mRNA for CCR3, CCR4, CCR8, and CCR9 were low in all of the three groups of mice, and they did not differ between the groups (Fig. 3B). Expression levels of CCR6 were found to be lower in the recipients of either WT or Prf1^−/−^ CD8^+^ immune T cells than in the control animals without the T cell transfer (Fig. 3B). These results indicate that CXCR3 and CXCR6 are unique among the 15 chemokine receptors tested and that their mRNA levels increased only when the perforin-dependent, CD8^+^ T cell-mediated anticyst immune process was operating against T. gondii cysts.

There are three chemokines, CXCL9, CXCL10, and CXCL11, which bind CXCR3. Amounts of mRNA for each of these three chemokines were markedly greater in the SCID mice that had received either WT or Prf1^−/−^ CD8^+^ immune T cells than in the control mice that received no T cells (Fig. 3C). There were no differences in the mRNA levels for each of these chemokines between the WT and Prf1^−/−^ T cell recipients (Fig. 3C). Amounts of CXCL16, the ligand for CXCR6, were also greater in the recipients of either WT or Prf1^−/−^ CD8^+^ immune T cells than in the control mice without the T cell transfer, and there were no differences in its mRNA levels between these two T cell recipient groups (Fig. 3C). The detection of increased levels of mRNA for the ligands for each of CXCR3 and CXCR6 suggests that both of these two chemokine receptors can function in the brains of the recipients of the immune T cells. Both CXCR3 and CXCR6 can be expressed on microglia and macrophages (16–19). Since our recent studies demonstrated that both Iba1^+^ microglia and Ly6C^+^ inflammatory macrophages accumulate to T. gondii cysts during CD8^+^ cytotoxic T cell-mediated elimination of the cysts (13), it is possible that CXCR3 and CXCR6 mediate the accumulation of these phagocytes to the sites of anticyst immune responses once CD8^+^ T cells initiate this process through their perforin-mediated activities.

### Molecules related to an activation of microglia and macrophages were upregulated in their mRNA levels in association with the CD8^+^ T cell-mediated removal of T. gondii cysts.

**FIG 4:**
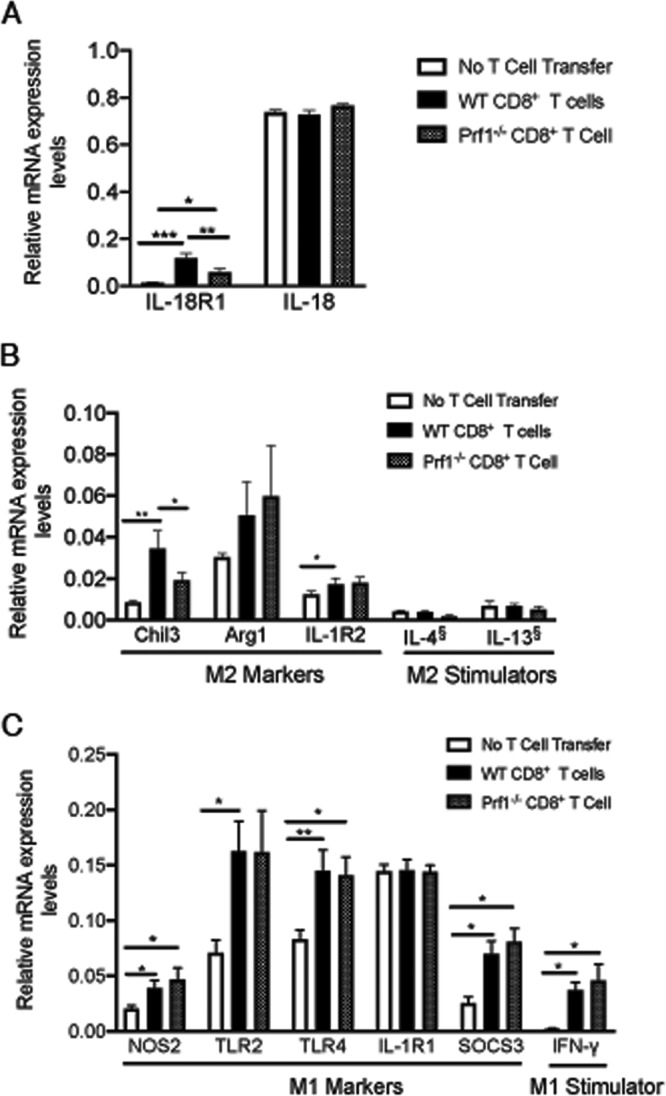
Differences in amounts of mRNA for molecules related to activation of microglia and macrophages in T. gondii-infected SCID mice that had received WT or Prf1^−/−^ CD8^+^ immune T cells and in control animals that had received no T cells. SCID mice were infected with 20 cysts of the ME49 strain of T. gondii and treated with sulfadiazine beginning at 9 days after the infection to develop tissue cysts of the parasite in their brains. Animals were injected intravenously from a tail vein with CD8^+^ immune T cells (2.1 × 10^6^ cells) from infected WT or Pfr1^−/−^ mice at 3 weeks after infection. As a control, one group of infected SCID mice did not receive any T cells. The data represent results determined 7 days after the T cell transfer. (A) mRNA levels for IL-18 and IL-18R1. (B) mRNA levels for M2 macrophage-inducing cytokines (IL-4 and IL-13) and M2 macrophage markers (Chil3, Arg1, and IL-1R2). (C) mRNA levels for an M1 macrophage-inducing cytokine (IFN-γ) and M1 macrophage markers (NOS2, TLR2, TLR4, IL1R1, and SOCS3). There were 3 to 5 mice in each experimental group. Data shown are means ± SEM. *, *P < *0.05; **, *P < *0.01. §, below the background value of the assay, which is the average of data from the negative-control transcripts.

IL-18 is a cytokine that activated microglia and macrophages can produce (22, 23). This cytokine can also activate both microglia and macrophages (22, 23). The receptor for this cytokine, IL-18R1, was among the six molecules whose cerebral mRNA expression levels were significantly greater in the infected SCID mice that had received WT CD8^+^ T cells than in those that had received Prf1^−/−^ CD8^+^ T cells (Fig. 1B). Expression levels of mRNA for both IL-18 and IL-18R1 in each of the three groups of infected SCID mice with and without the T cell transfer are shown in Fig. 4A. The IL-18R1 mRNA levels were 2.3 and 11 times greater in the brains of the WT T cell recipients than in those of Prf1^−/−^ T cell recipients and control mice with no T cell transfer, respectively (*P < *0.01 and *P < *0.001, respectively, Fig. 4A). High levels of IL-18 mRNA were detected in the brains of all of these three groups of mice, and there were no differences in its expression levels between these groups (Fig. 4A). These results suggest that the greater expression of IL-18R1 in the recipients of WT CD8^+^ immune T cells can result in greater activation of microglia and macrophages expressing IL-18R1 during perforin-dependent elimination of T. gondii cysts mediated by the T cells.

Activated macrophages are often classified into classically activated (M1) types and alternatively activated (M2) types based on their functions and expression of marker molecules (24, 25). A typical activator of M1 macrophages is IFN-γ, whereas typical activators of M2 macrophages are IL-4 and IL-13 (24, 25). Common markers for M1 macrophages include inducible nitric oxide synthase 2 (NOS2) and Toll-like receptor 2 (TLR2), TLR4, IL-1R1, and suppressor of cytokine signaling 3 (SOCS3). Markers for M2 macrophages include arginase 1 (Arg1), Chil3, and IL-1R2. Whereas Chil3 was one of the six molecules whose mRNA levels were significantly upregulated in the brains of the recipients of WT CD8^+^ immune T cells compared to the recipients of Prf1^−/−^ CD8^+^ T cells as well as to the no-T cell transfer controls (Fig. 1B), mRNA levels for the other M2 markers, Arg1 and IL-1R2, did not differ between the recipients of the WT and Prf1^−/−^ CD8^+^ immune T cells (Fig. 4B). Consistently, mRNA levels for the M2-inducing cytokines, IL-4 and IL-13, were low and below the background value of the assay in all of the three experimental groups (Fig. 4B). In contrast to those for the M2 markers, mRNA levels for all of the M1 markers, NOS2, TLR2, TLR4, IL1R1, and SOCS3, were high in the recipients of both the WT and Prf1^−/−^ CD8^+^ immune T cells, and the expression levels did not differ between these two groups (Fig. 4C). Consistently, increased levels of IFN-γ mRNA were detected in both of these recipient groups, and their expression levels did not differ between these groups (Fig. 4C). Therefore, it appears that the upregulation of Chil3 during the perforin-dependent elimination of T. gondii cysts is not due to an induction of M2 macrophages.

### Upregulation of ICOS, CXCR3, CXCR6, and IL-18R in the brains of T. gondii-infected SCID mice following a transfer of CD8^+^ immune T cells compared to a transfer of the normal CD8^+^ T cells.

We previously demonstrated that in contrast to a transfer of CD8^+^ immune T cells, a transfer of CD8^+^ normal T cells from uninfected WT mice into infected SCID mice did not reduce the cyst burden in their brains within 1 week after the cell transfer (12, 26). Therefore, we examined whether cerebral mRNA levels for ICOS, ICOSL, CXCR3, CXCR6, IL-18R1, and Chil3 differed between the brains of SCID mice following a transfer of CD8^+^ immune and normal T cells from infected and uninfected WT mice, respectively, as observed between the WT and Prf1^−/−^ CD8^+^ immune T cells shown in Fig. 1 to 4. SCID mice infected and treated with sulfadiazine received CD8^+^ T cells (7.5 × 10^6^ cells) from uninfected or infected WT mice at 3 weeks after infection. As a control, a group of SCID mice did not receive any T cells. Amounts of mRNA for the six molecules in the brains of these three groups of mice were measured by real-time RT-PCR at 7 days after the cell transfer.

**FIG 5:**
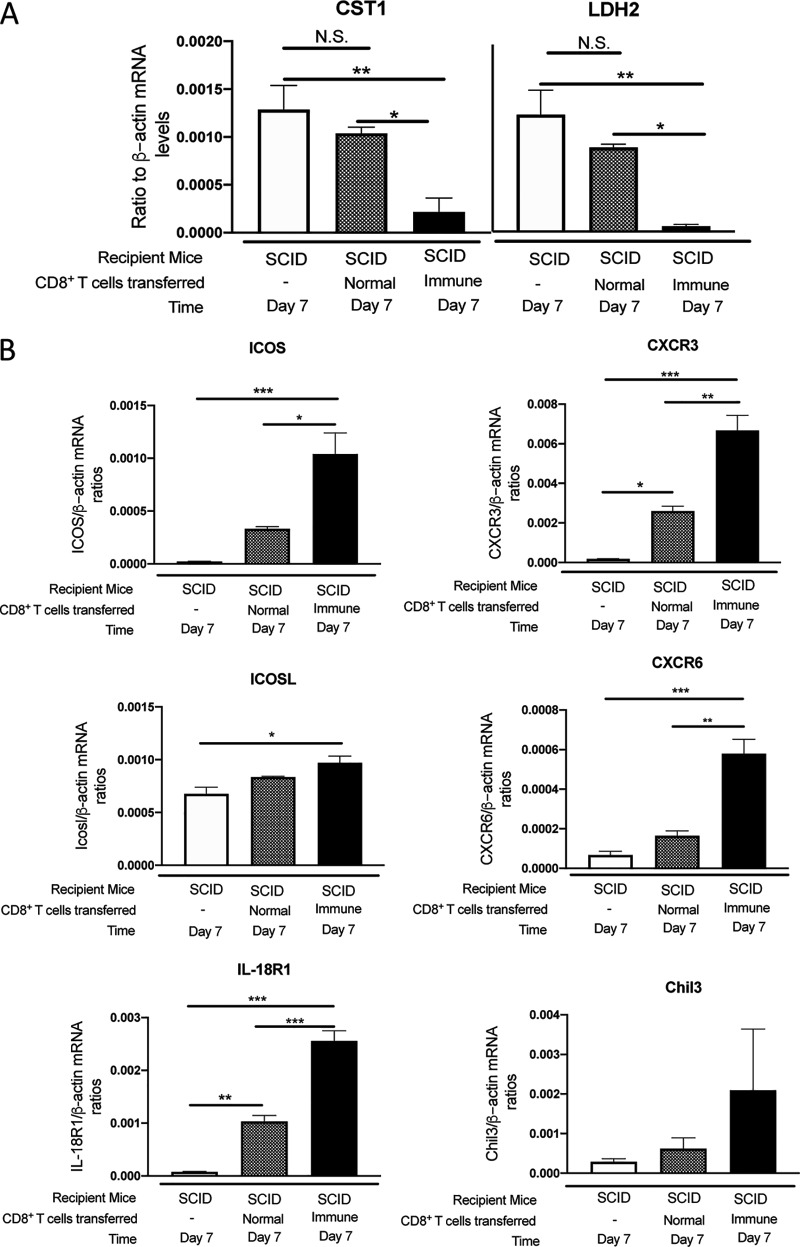
Differences in amounts of mRNA for ICOS, ICOSL, CXCR3, CXCR6, IL-18R, and Chil3 in T. gondii-infected SCID mice that had received WT CD8^+^ immune or normal T cells. SCID mice were infected with 20 cysts of the ME49 strain of T. gondii and treated with sulfadiazine beginning at 9 days after the infection to induce formation of tissue cysts of the parasite in their brains. The animals in one group were injected intravenously from a tail vein with CD8^+^ immune T cells (7.5 × 10^6^ cells) from infected WT mice at 3 weeks after infection. The mice in another group received normal CD8^+^ T cells from uninfected WT mice in the same manner. As an additional control, one group of infected SCID mice did not receive any T cells. The data represent results determined 7 days after the T cell transfer. (A) mRNA levels for bradyzoite (cyst)-specific CST1 and LDH2. (B) mRNA levels for ICOS, ICOSL, CXCR3, CXCR6, IL-18R, and Chil3. There were 4 mice in each experimental group. Data shown are means ± SEM. *, *P < *0.05; **, *P < *0.01; ***, *P < *0.001.

Cyst burdens measured by amounts of mRNA for bradyzoite-specific CST1 and LDH2 were significantly lower in the brains of WT CD8^+^ immune T cell recipients than in those of the control animals without the T cell transfer (*P < *0.01, Fig. 5A). In contrast, amounts of mRNA for these bradyzoite-specific molecules in the normal CD8^+^ T cell recipients did not differ from those in the controls (Fig. 5A). In addition, both CST1 and LDH2 mRNA levels in the CD8^+^ immune T cell recipients were significantly lower than in the normal T cell recipients (*P < *0.05, Fig. 5A). These results confirmed that a transfer of CD8^+^ normal T cells is not capable of reducing T. gondii cyst burden as quickly as the immune T cells do following their transfer into infected SCID mice.

Amounts of mRNA for ICOS, CXCR3, CXCR6, and IL-18R1 in the brains of the WT immune T cells were significantly greater than in those of the WT normal T cells as well as those of the control mice without any T cell transfer (Fig. 5B), which was consistent with the results shown in Fig. 1 to 4. The mRNA levels for ICOSL in the immune T cell recipients were greater than those in the controls without any T cell transfer (*P* < 0.05, Fig. 5B) and also tended to be greater than those in the normal T cell recipients. However, the difference between the immune and normal T cell recipients did not reach significance (Fig. 5B). Expression levels of Chil3 mRNA also showed a similar tendency, with the highest expression levels seen in the immune T cell recipients, but the differences did not reach statistical significance mostly due to large variations in the value among individual mice in the immune T cell recipients (Fig. 5B).

### Capability of CD8^+^ immune T cells to eliminate T. gondii cysts that persisted in the brains of infected mice until 6 weeks after infection.

**FIG 6:**
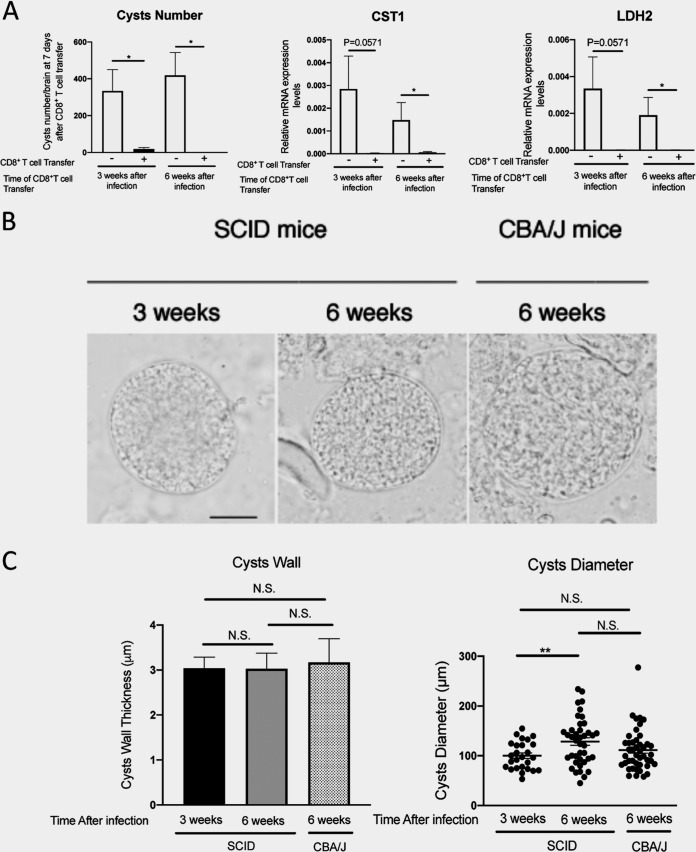
CD8^+^ T cell-mediated elimination of T. gondii cysts in the brains of SCID mice that had been infected for 6 weeks. Four groups of SCID mice were infected with 20 cysts of the ME49 strain of T. gondii and treated with sulfadiazine beginning at 9 days after the infection to induce formation of tissue cysts of the parasite in their brains. At 3 weeks after infection, the animals in one group were injected intravenously from a tail vein with CD8^+^ immune T cells (1.8 × 10^6^ cells) from infected WT mice, and the mice in another group did not receive any T cells as a control. At 6 weeks after infection, one of the other two groups of the animals received CD8^+^ immune T cells from infected WT mice in the same manner, and another group did not receive any T cells as a control. The data represent results determined 8 days after each of the T cell transfers. For comparing the morphology of the cysts in the brains of SCID mice at 3 and 6 weeks after infection, CBA/J mice were infected with 10 cysts using the same cyst preparation that was used for infecting the SCID mice. Cysts in the brains of the CBA/J mice were examined at 6 weeks after infection. (A) Numbers of T. gondii cysts and mRNA levels for bradyzoite (cyst)-specific CST1 and LDH2 in the brains of SCID mice that had received the CD8^+^ immune T cells at either 3 or 6 weeks after infection. (B) Images of representative T. gondii cysts detected in the brain suspensions of SCID mice that had been infected for 3 or 6 weeks and of a brain suspension of CBA/J mice that had been infected for 6 weeks. (Left panel) Bar, 50 μm. All images are at the same magnification. (C) (Left panel) The thickness of cyst wall in the cysts detected in brain suspensions from SCID mice infected for 3 or 6 weeks and CBA/J mice infected for 6 weeks. Eight to 14 cysts from each mouse in each group (*n* = 3 or 4) were analyzed. (Right panel) Diameters of the cysts detected in brain suspensions from SCID mice infected for 3 or 6 weeks and CBA/J mice infected for 6 weeks. Each dot indicates the size of each single cyst in the group. Eight to 13 cysts from each mouse in each group (*n* = 3 or 4) were analyzed. In panel A and in the graph at the left in panel C, data shown are means ± SEM. In the graph at the right in panel C, each bar indicates the mean value in the group. *, *P < *0.05; **, *P < *0.01.

Since a transfer of CD8^+^ immune T cells was performed in SCID mice at 3 weeks after infection in all the experiments shown in Fig. 1 to 5, it may be argued that the CD8^+^ cytotoxic T cells are able to eliminate only early-stage cysts of T. gondii which have been formed recently. Therefore, we examined whether a transfer of CD8^+^ immune T cells (1.8 × 10^6^ cells) would be able to remove the cysts from the brains of SCID mice that had been infected for 6 weeks. As a negative control, a group of infected SCID mice received no T cells. In comparison with these two groups of mice, a group of SCID mice received the immune T cells (1.8 × 10^6^ cells) at 3 weeks after infection in the same manner as in the studies described for Fig. 1 to 5. At 8 days after the T cell transfer to the SCID mice that had been infected for 6 weeks, the numbers of T. gondii cysts in their brains were markedly lower than were detected in the brains of the negative-control mice without the T cell transfer (*P < *0.05, Fig. 6A). The reduction of cyst numbers in the T cell recipients was marked and seemed even more marked than that detected after the T cell transfer to SCID mice that had been infected for 3 weeks (Fig. 6A). Consistently, the amounts of mRNA for bradyzoite-specific CST1 and LDH2 were dramatically lower in the CD8^+^ T cell recipients than in the controls following the T cell transfer at 6 weeks after infection (*P < *0.05, Fig. 6A). Marked reductions of mRNA levels for these two bradyzoite-specific molecules were also detected following the T cell transfer at 3 weeks after infection, as expected (Fig. 6A), although the *P* values determined in statistical analyses of comparisons between the T cell recipients and the controls were a little over 0.05 due to larger variations of the values in the control group (Fig. 6A). These results indicate that CD8^+^ immune T cells are capable of eliminating not only the T. gondii cysts that had been formed in a few weeks but also those that had persisted 6 weeks after infection.

Since the thickness of the cyst wall is an indicator of the maturity of T. gondii cysts, we compared the thickness of the cyst wall of the cysts detected in the brain suspensions of SCID mice that had been infected for 3 or 6 weeks to that of the cysts in the brain suspensions of immunocompetent CBA/J mice that had been infected for 6 weeks. Representative images of the cysts detected in all three groups of mice are shown in Fig. 6B. The thickness of the cyst wall was measured for 8 to 14 cysts from each mouse in each group that had 3 or 4 mice. The cyst wall thickness of these cysts was around 3 μm in the brains of the SCID mice at both 3 and 6 weeks after infection (Fig. 6C, left panel). In addition, the wall thickness of the cysts in each of these two groups of infected SCID mice was equivalent to that of the cysts detected in the brains of CBA/J mice at 6 weeks after infection (Fig. 6C, left panel), indicating that the cysts present in the brains of infected SCID mice at both 3 and 6 weeks after infection were mature cysts equivalent to those present in immunocompetent CBA/J mice that had been infected for 6 weeks. This point was further supported by the evidence indicating that the diameters of the cysts detected in the brains of SCID mice at both 3 and 6 weeks after infection did not differ from those of the cysts detected in the brains of CBA/J mice at 6 weeks after infection (Fig. 6C, right panel), although the diameters of the cysts in the SCID mice at 3 weeks after infection were slightly smaller than those in the SCID mice at 6 weeks after infection (*P < *0.01, Fig. 6C, right panel). These results reflecting the morphology of the cysts along with the potent capability of CD8^+^ T cells to eliminate T. gondii cysts from the brains of SCID mice that had been infected not only for 3 weeks but also for 6 weeks indicate that the CD8^+^ immune T cells are able to eliminate mature cysts of T. gondii from the brains of infected mice.

### Upregulation of ICOS, CXCR3, CXCR6, IL-18R1, and Chil3 in the brains of T. gondii-infected SCID mice that received a transfer of CD8^+^ immune T cells at 6 weeks after infection.

**FIG 7:**
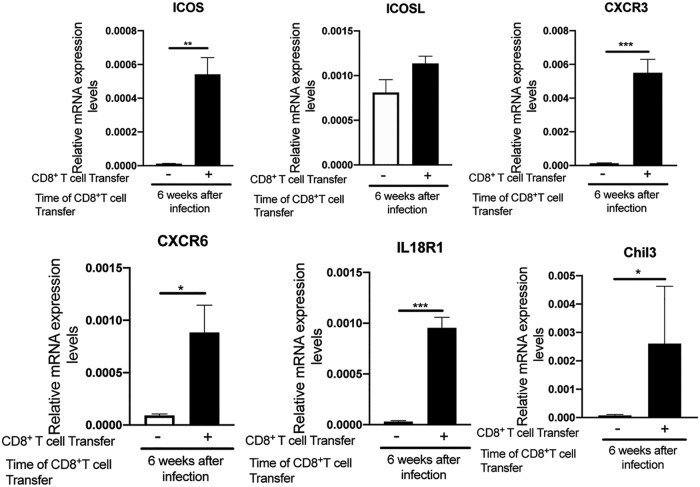
Differences in amounts of mRNA for ICOS, ICOSL, CXCR3, CXCR6, IL-18R1, and Chil3 between T. gondii-infected SCID mice that had received WT CD8^+^ immune T cells at 6 weeks after infection and those that had not received any T cells. SCID mice were infected with 20 cysts of the ME49 strain of T. gondii and treated with sulfadiazine beginning at 9 days after the infection to induce formation of tissue cysts of the parasite in their brains. At 6 weeks after infection, the animals in one group were injected intravenously from a tail vein with CD8^+^ immune T cells (1.8 × 10^6^ cells) from infected WT mice, and the mice in another group did not receive any T cells as a control. The data represent results determined 8 days after the T cell transfer. There were 3 or 4 mice in each experimental group. Data shown are means ± SEM. *, *P < *0.05; **, *P < *0.01; ***, *P < *0.001.

We next examined whether mRNA expression levels for ICOS, ICOSL, CXCR3, CXCR6, IL-18R1, and Chil3 increased during the removal of T. gondii cysts following a transfer of CD8^+^ immune T cells into SCID mice that had been infected for 6 weeks. The amounts of mRNA for ICOS, CXCR3, CXCR6, IL-18R1, and Chil3 were significantly greater in the brains of SCID mice that had received the immune T cells than in the control animals that had received no T cells (*P < *0.05, *P < *0.01, or *P < *0.001, Fig. 7). ICOSL mRNA levels also tended to be greater in the T cell recipients than in the controls, but the difference was relatively small and did not reach statistical significance (Fig. 7). These results were consistent with those shown in Fig. 5B, representing a study in which the T cell transfer was performed at 3 weeks after infection in SCID mice. Therefore, these molecules, ICOS, ICOSL, CXCR3, CXCR6, IL-18R1, and Chil3, most likely play important roles in the CD8^+^ T cell-dependent protective immune process for eliminating mature T. gondii cysts.

## DISCUSSION

The present study revealed that the CD8^+^ immune T cells were able to eliminate the majority of T. gondii cysts from the brains of not only the SCID mice that had been infected for 3 weeks but also the mice that had been infected for 6 weeks. In addition, the thickness of the cyst wall and sizes of the cysts, indicators of the maturity of this chronic-stage form of T. gondii, detected in the brains of infected SCID mice at both 3 and 6 weeks after infection were equivalent to those of the cysts detected in the brains of WT CBA/J mice that had been infected for 6 weeks, the time period considered to be sufficient for T. gondii cysts to mature. This is consistent with the evidence from our previous study indicating an occurrence of an association of CD8^+^ T cells with T. gondii cysts regardless of the cyst sizes in the brains of infected mice (13). Thus, CD8^+^ cytotoxic T cells have a potent capability to eliminate mature T. gondii cysts from the brains of chronically infected hosts.

The present study uncovered that mRNA expression levels for only six selected molecules, ICOS, ICOSL, CXCR3, CXCR6, IL-18R1, and Chil3, among 734 genes related to immune responses are upregulated specifically in the presence of perforin-dependent, CD8^+^ immune T cell-mediated protective immune responses to eliminate T. gondii cysts in the brain of infected mice compared to the presence of perforin-deficient CD8^+^ immune T cells. Significant increases in mRNA levels for ICOS, CXCR3, CXCR6, IL-18R1, and Chil3 were detectable during cyst elimination by perforin-dependent anticyst activity of CD8^+^ T cells initiated at 6 weeks after infection. Significant increases in ICOSL mRNA levels were also detected during the CD8^+^ T cell-mediated removal of T. gondii cysts initiated at 3 weeks after infection. Therefore, these six molecules, ICOS, ICOSL, CXCR3, CXCR6, IL-18R1, and Chil3, appear to play important roles in the cytotoxic T cell-mediated anticyst immune process to eliminate T. gondii cysts, and the effector mechanisms in which these six molecules are involved are quite effective even against mature cysts of this parasite.

ICOS and ICOSL were among those six molecules selectively activated during the perforin-dependent anticyst immune process. ICOS and ICOSL are known to be among the T cell costimulatory molecule systems along with CD137 (4-1BB) and CD137L (4-1BBL) as well as the CD28 and CD80/CD86 costimulatory systems. Although the interactions between CD137 and CD137L are critical for CD8^+^ T cell activation, the importance of the ICOS-ICOSL system in CD8^+^ T cell responses has also been recognized (14, 15). ICOS is expressed on activated CD8^+^ T cells in addition to CD4^+^ T cells, and the ICOS costimulation is important for CD8^+^ T cell expansion following their stimulation (14). Importantly, retrogenic ICOS expression increases the cytotoxic capability of CD8^+^ T cells, whereas it reduces their capability of producing IFN-γ, IL-2, and tumor necrosis factor alpha (TNF-α) (15). The results from the present study, along with the evidence from those previous studies, suggest that the ICOS-ICOSL costimulation plays an important role in activation and expansion of T. gondii-specific CD8^+^ cytotoxic T cells to effectively carry out the perforin-dependent immune process to remove the tissue cysts of T. gondii from the brain.

The present study demonstrated that two chemokine receptors, CXCR3 and CXCR6, are among the six molecules whose expression specifically increases during the perforin-dependent elimination of T. gondii cysts by CD8^+^ T cells in the brain. Both CXCR3 and CXCR6 can be expressed on microglia and macrophages (16–19). Our previous studies depicted an accumulation of large numbers of Iba1^+^ microglia and Ly6C^+^ inflammatory macrophages during CD8^+^ cytotoxic T cell-mediated removal of T. gondii cysts in the brains of infected mice (13). In addition, bradyzoites located in the destroyed cysts were detected within the accumulated microglia and macrophages (13), and an inhibition of phagolysosomal acidification by chloroquine inhibited, at least in part, the CD8^+^ T cell-mediated elimination of the cysts (27). Since the ligands for these chemokine receptors, CXCL9, CXCL10, and CXCL11 for CXCR3 and CXCL16 for CXCR6, are highly expressed in the brains of infected mice during the cyst elimination as shown in Fig. 3C, it is highly possible that CXCR3 and CXCR6 expressed on the surface of microglia and macrophages play a crucial role in mediating their migration to T. gondii cysts, when under attack by CD8^+^ cytotoxic T cells, for killing and removal of bradyzoites located within the cysts.

IL-18R1 is another molecule whose expression was upregulated in association with the perforin-dependent, CD8^+^ T cell-mediated removal of T. gondii cysts in the present study. Both microglia and macrophages can produce IL-18 and respond to this cytokine through IL-18R1 expressed on their surface (22, 23). In addition, IL-18 enhances the phagocytic activities of both microglia (20) and macrophages (21). Since IL-18 mRNA levels were equally upregulated in the brains of infected SCID mice that had received either perforin-sufficient or perforin-deficient CD8^+^ immune T cells, an activation of microglia and macrophages by binding of IL-18 to IL-18R1 expressed on their surface most likely plays an important role in enhancing their phagocytic activities to eradiate T. gondii bradyzoites located within the cysts during the CD8^+^ cytotoxic T cells-mediated anticyst immune process. This possibility is supported by the evidence from our recent study, an involvement of phagolysosomal acidification in the CD8^+^ T cell-mediated elimination of the cysts (27).

Chil3 is also among the six molecules identified by the present study whose transcript levels increased during CD8^+^ cytotoxic T cell-mediated elimination of T. gondii cysts. Although Chil3 is often described as one of markers for M2-type macrophages (28, 29), the present study showed that mRNA levels of the M2-inducing cytokines IL-4 and IL-13 were quite low regardless of the presence or absence of perforin-dependent anticyst immunity. Furthermore, the macrophage marker phenotypes seen during the anticyst immune process more closely resembled those of the M1 type than the M2 type. Therefore, it is possible that the increased levels of Chil3 mRNA detected during the elimination of T. gondii cysts were the consequence of a collaboration of CD8^+^ cytotoxic T cells and phagocytes and did not indicate an involvement of M2-type macrophages and microglia. Chil3 is most likely involved in the protective activities in a different manner(s).

The profiling of transcript expression for 734 genes related to immune responses using a NanoString assay effectively identified six molecules selectively upregulated during the immune process of T. gondii cyst removal mediated by perforin-dependent activities of CD8^+^ immune T cells in collaboration with an accumulation of microglia and macrophages. Elucidating how these six molecules contribute to the collaboration between the cytotoxic T cells and phagocytes will provide valuable insights into a novel effector pathway of the immune system mediated by an invasion of CD8^+^ cytotoxic T cells into a large target, T. gondii cysts, with an amplification of the effector capability by an accumulation of large numbers of phagocytes.

### Summary.

The present study, employing large-scale transcript analysis of immunity-related genes, uncovered that the mRNA expression levels for 6 genes, *Icos*, *IcosL*, *Cxcr3*, *Cxcr6*, *IL18r1*, and *Chil3*, among 734 genes analyzed selectively increased in a manner that depended on the presence of perforin specifically in CD8^+^ immune T cells during the operation of the protective immunity to eliminate a large target, T. gondii cysts, in the brain of mice infected with the parasite. ICOS and ICOSL are the molecules involved in T cell activation. CXCR3, CXCR6, and IL-18R1 are the molecules that can induce migration and activation of phagocytes, which accumulate around and migrate into the target during the CD8^+^ cytotoxic T cell-mediated elimination of the target. Since cytotoxic T cells play important roles in resistance against various microorganisms and cancers, the information generated by the present study could be helpful to improve our understanding of the mechanisms of the host immunity not only against T. gondii but also these other targets, especially those consisting of large masses such as solid cancers.

## MATERIALS AND METHODS

### Mice.

Swiss-Webster mice were obtained from Taconic (Germantown, NY). BALB/c, CBA/J, and BALB/c-background SCID mice were from Jackson Laboratories (Bar Harbor, ME). BALB/c-background Prf1^−/−^ mice (30) were originally provided by John T. Harty and were bred in our animal facility. Age-matched female mice were used for all the studies. Mice were housed in a pathogen-free environment, and experimental procedures were performed in sterile settings. There were 3 to 5 mice in each experimental group. All animal procedures were approved by our Institutional Animal Care and Use Committee.

### Infection with Toxoplasma gondii.

Cysts of the ME49 strain of T. gondii were obtained from the brains of Swiss-Webster mice infected intraperitoneally with 10 cysts. WT BALB/c, CBA/J, and Prf1^−/−^ mice were infected orally by gavage with 10 cysts. SCID mice were infected orally by gavage with 20 cysts and treated with sulfadiazine (400 mg/liter) in their drinking water beginning at 9 days after infection for the entire period of the experiment to control the proliferation of tachyzoites and establish a chronic infection. The infected Prf1^−/−^ mice also received sulfadiazine beginning at 26 days after infection.

### Purification of CD8^+^ T cells and their transfer into infected SCID mice.

Spleen cells obtained from chronically infected Prf1^−/−^ and WT mice were suspended in Hanks’ balanced salt solution (Irvine Scientific, Santa Ana, CA) with 2% fetal bovine serum (Sigma, St. Louis, MO). CD8^+^ T cells were purified from the spleen cells using magnetic-bead-conjugated anti-mouse CD8 (clone 53-6.7) monoclonal antibodies (Miltenyi Biotech, Auburn, CA) and a MACS column (Miltenyi). A total of 2.1 × 10^6^ cells of CD8^+^ T cells were injected intravenously from the tail into infected SCID mice at 3 weeks after infection. As a control, one group of infected SCID mice did not receive any T cells. In another experiment, infected SCID mice received 7.5 × 10^6^ cells of CD8^+^ normal T cells purified from the spleen of uninfected WT mice or CD8^+^ immune T cells from infected WT mice in the same manner. One control group without transfer of any T cells was also included. To confirm the capability of CD8^+^ immune T cells to eliminate cysts that had persisted for more than a few weeks, in another experiment, infected and sulfadiazine-treated SCID mice received 1.8 × 10^6^ cells of CD8^+^ immune T cells at 3 or 6 weeks after infection. A group that received no T cells was included for each of the two time points as a control.

### RNA extraction and NanoString gene expression analysis.

The brains were obtained from each experimental group at 7 or 8 days after a transfer of CD8^+^ T cells. RNA extraction from the brain was performed as described previously (12, 31). RNAs were isolated from half of the brain of each of the SCID mice using RNA STAT-60 (Tel-Test, Friendswood, TX). The RNA concentration was measured using a NanoDrop ND-2000 spectrophotometer (Thermo Fisher Scientific, Waltham MA). Total RNA (100 ng) from the half-brain of each of the mice was quality checked using a Bioanalyzer for an RNA integrity score (RIN) value of >9.0. The quality-confirmed RNA was then used to detect 734 gene expression levels using a NanoString nCounter mouse myeloid innate immunity panel (NanoString Technologies, Seattle, WA). The assay was performed by the Genomics Core Laboratory at the University of Kentucky. Data were analyzed using nCounter and nSolver software (NanoString Technologies) and were normalized using positive-control and housekeeping genes. Transcript data on certain genes were excluded from analysis when their transcript counts were lower than the background value of the assay, which represented the average of data from the negative-control transcripts.

### Real-time RT-PCR.

Real-time RT-PCR was performed as described previously (12, 31). The purified RNA was first treated with DNase I (Invitrogen, Waltham, MA) to remove genomic DNA contamination. cDNA was synthesized with 1 μg of the DNase I-treated RNA using reverse transcriptase (Invitrogen) and random hexamers (Invitrogen). The PCRs were performed with the cDNA using a StepOnePlus real-time PCR system with TaqMan reagents (Applied Biosystems, Branchburg, NJ). All primers and probes were from Applied Biosystems. The reagents used for ICOS, ICOSL, CXCR3, CXCR6, IL-18R1, and Chil3 were their ready-made products. The sequences of primers and probe for T. gondii bradyzoite (cyst)-specific CST1 were previously described (13). The sequences of primers and probe for another bradyzoite-specific molecule, lactate dehydrogenase (LDH2), were as follows: forward primer 5′→3′, TTTGTGTGCTTCGGGAGCTA; reverse primer 5′→3′, ATCCAACGCCTTCCCTTCTG, probe 5′→3′, CATGCCTGTTACAACGTC. Amounts of mRNA for these molecules were normalized to amounts of mRNA for a housekeeping gene, the β-actin gene.

### Numbers and morphology of cysts in the brains of infected mice.

A half-brain of each of SCID mice at 3 or 6 weeks after infection was homogenized using a mortar and pestle with 0.5 ml of phosphate-buffered saline (PBS). Homogenates of the brains of CBA/J mice were prepared in the same manner at 6 weeks after infection. Numbers of T. gondii cysts in two aliquots (20 μl each) were microscopically counted. Images of 8 to 14 cysts from each brain were also taken at 40× magnification using Nikon Eclipse 90*i* microscope equipped with a digital camera. The thickness of the cyst wall and diameter of each cyst were determined using NISElements BR acquisition 3.2 (Nikon). At 7 or 8 days after the CD8^+^ T cell transfer, numbers of the cysts in the brains of recipient SCID mice were determined in the same manner.

### Statistical analysis.

Levels of statistical significance of results of comparisons between experimental groups were determined by one-way analysis of variance (ANOVA) followed by Turkey *post hoc* analysis or by Student's *t* test using GraphPad Prism 8.0 software (GraphPad, La Jolla, CA). When the latter was used, corrected *P* (*Pc*) values were calculated by multiplying the *P* value by the number of comparisons performed in the analysis. Differences in expression levels of molecules in volcano plots calculated from NanoString data were determined using Prism 8.0 software. In this analysis, the statistical significance value for each molecule was adjusted by the total number of molecules tested in the assay. Differences that provided *P* values of <0.05 or *Pc* values of <0.05 were considered significant.
